# Relational and Communication Strategies Utilized by Underserved Care Partners and Family Members with Dementia

**DOI:** 10.1093/geroni/igaf122.2232

**Published:** 2025-12-31

**Authors:** Carissa Coleman, Amanda Runyan

**Affiliations:** University of Kansas Medical Center, Kansas City, Kansas, United States; Robert J. Dole VA Medical Center, Wichita, Kansas, United States

## Abstract

Dementia burden is disproportionally carried by underserved communities resulting in higher intensity in-home caregiving and greater unmet needs. Communication is a challenge for family dementia care dyads and underserved ADRD populations face greater challenges due to lack of access. Identifying evidence for strategies facilitating or impeding communication and gaps in education and technology needs is needed for an inclusive communication intervention. This study expands on our existing communication research, extending to a diverse sample to understand their unique experiences. Ten, one-hour, mixed-method interviews were conducted with dementia care dyads. Eight dyads were African American and two were Hispanic/Latino. All care partners were female (daughter or spouse). The people with dementia were mostly female (80%) with Alzheimer’s (50%) categorized as severe (40%). Operational definitions for relational strategies and verbal/nonverbal communication behaviors were developed in Dedoose and inter-rater reliability was excellent (Kappa = .86, .81). During analysis of the transcripts, more complex strategies were identified. The most common relational strategies included personalized care (29.6%), managing emotions (27.2%), and tending to the environment (20.2%). Orienting (34.6%) and clarifying (23.4%) were the most common verbal strategies. Utilizing time (25.4%) and the environment (22.4%) were the most common nonverbal strategies. Preferred educational modalities were those that facilitated face-to-face connections with others versus technology-based modalities. In-interview survey responses were notably different than spontaneous responses indicating dementia dyads identified their own strategies without prompting. Based on these findings, family dementia care communication education will be developed tailored to the needs of diverse dementia dyads and their communities.

